# A small-molecule RGD-integrin antagonist inhibits cell adhesion, cell migration and induces anoikis in glioblastoma cells

**DOI:** 10.3892/ijo.2012.1708

**Published:** 2012-11-20

**Authors:** MARIKA A. RUSSO, MAYRA PAOLILLO, YULY SANCHEZ-HERNANDEZ, DANIELA CURTI, EMILIO CIUSANI, MASSIMO SERRA, LINO COLOMBO, SERGIO SCHINELLI

**Affiliations:** 1 Department of Developmental and Molecular Biology, Albert Einstein College of Medicine of Yeshiva University, Bronx, NY 10461, USA;; 2 Departments of Drug Sciences and; 3 Biology and Biotechnology ‘Lazzaro Spallanzani’, University of Pavia, I-27100 Pavia;; 4 Carlo Besta Neurological Institute, I-20133 Milan, Italy

**Keywords:** glioblastoma, integrins, migration, anoikis

## Abstract

In cancer cells integrins modulate important cellular events that regulate the metastasic cascade which involves detachment from the tumor mass, dissemination and attachment to the oncogenic niche. The α5β1, αvβ3 and αvβ5 integrins are widely expressed in different cancer types and recognize the tripeptide Arg-Gly-Asp (RGD) motif present in several extracellular matrix proteins. In human glioblastoma, αvβ3 integrin expression correlates with tumor grade, suggesting that this integrin may play a crucial role in the highly infiltrative behavior of high grade gliomas. However, few selective RGD-like antagonists have been developed and few studies have investigated their effects in *in vitro* models of human glioblastoma. In this study, we investigated several cellular effects and the underlying molecular mechanisms exerted by a new small-molecule RGD antagonist, 1a-RGD, in the U251 and U373 human glioblastoma cell lines. Treatment with 1a-RGD (20 *μ*M) demonstrated a weak effect on cell viability and cell proliferation but strongly inhibited cell attachment and cell migration together with actin cytoskeleton disassembly. Prolonged 1a-RGD treatment (72 h) induced anoikis, assessed by Annexin staining and nucleosome assay, particularly in the detached cells. When integrin-linked transduction pathways were investigated, 1aRGD was found to exert a marked reduction in focal adhesion kinase (FAK) phosphorylation without affecting the AKT- and ERK-dependent pathways. Our data indicate that 1a-RGD, probably via modulation of the FAK-dependent pathway, inhibits cell migration and attachment and induces anoikis in glioblastoma cells. This novel finding suggests that the development of an RGD-like molecule may represent a promising tool for the pharmacological approach aimed at reducing the malignancy of glioblastoma cells.

## Introduction

Human glioblastoma is one of the most lethal cancer types and, in spite of the mass of *in vitro* and *in vivo* studies accumulated in the literature, currently used standard therapies still result in a median duration of patient survival of 12–18 months after diagnosis. The key objective to improve glioblastoma pharmacological therapy lies in the ability to prevent the dissemination of single cancer cells that eventually contributes to the reformation of new solid tumor masses.

The invasiveness of brain cancer cells is a complex mechanism that involves several steps such as initial detachment of tumorigenic cells from the tumor mass, migration through brain parenchyma, resistance to apoptotic damage and finally adhesion to distal cells in the tumoral niche. The endogenous extracellular matrix (ECM) proteins, such as laminin, collagens, tenascin and vitronectin, play a fundamental role in cancer cell invasiveness since their binding to integrins modulates cell attachment and other processes such as proliferation and migration.

Integrins are heterodimeric glycoprotein membrane receptors formed by the combination of α and β subunits that give rise to 24 distinct integrins whose subunit composition leads to their ECM ligand specificity. The α5β1, αvβ3 and αvβ5 integrins, recognizing the tripeptide sequence Arg-Gly-Asp (RGD) present in many ECM proteins (1), are actively exploited as potential targets in the development of antitumorigenic and antiangiogenic compounds as they are overexpressed in glioma and peritumoral endothelial cells (2).

The binding of ECM ligands to integrins activates the cytosolic tyrosine kinase Src, constitutively bound to the integrin β cytoplasmic tail and the focal adhesion kinase (FAK) (3) that, in turn, leads to the activation of downstream ERK-and AKT-dependent signaling pathways.

FAK appears to play key roles in tumor growth and metastatic spread. It is overexpressed in glioblastoma tumor biopsy samples. It modulates proliferation, survival and migration of glioblastoma cells *in vitro* and in animal model (4) and its activation, mediated by integrin-ECM ligands, provides essential survival signals and protects glioma cells from anoikis, a detachment-induced cell death. For these reasons, inhibition of FAK activity is an appealing target.

Resistance to anoikis confers a selective advantage for tumor cell invasion and metastasis; therefore, reducing cancer cell dissemination by enhancing anoikis via integrin antagonists appears promising. However, although the validity of this hypothesis has been confirmed in different cancer cell types and endothelial cells with significant results (1), in glioma cells the complexity of the mechanisms involved in the induction and resistance to anoikis is a serious obstacle.

The first small molecule integrin antagonist developed was cilengitide (EMD 121974), a cyclic peptide belonging to the RGD-peptide family that, upon binding to the integrin β chain, prevents the interaction of integrins with their endogenous ECM ligands. Previous studies have demonstrated the promising features of RGD-peptide molecules, as these compounds display relative efficacy, good tolerability and low toxicity in clinical trials. Although cilengitide blocks glioblastoma (GBM) growth in nude mice (5), evidence in patients with recurrent GBM has shown that cilengitide monotherapy is well tolerated but displays modest antitumor activity (6). This finding has prompted efforts aimed at the synthesis of new peptidic and non-peptidic integrin antagonists with a different pattern of binding properties. These molecules are currently under investigation for their anti-angiogenic and anticancer activity, administered alone or in combination with other therapeutic agents such as temozolomide (7).

The new integrin antagonist 1a-RGD, unlike the cyclic peptide cilengitide, is an RGD-like molecule containing a bicyclic pseudopentapeptide that binds αvβ3, αvβ5 and α5β1 integrins with *in vitro* preferential affinity towards αvβ3. However, it is still unknown whether and how the novel chemical structure of 1a-RGD may interfere with the functional effects elicited by the ECM-integrin interaction in glioma cells *in vitro*.

In this study we evaluated several cellular effects induced by 1a-RGD treatment in human U251 and U373 glioblastoma cell lines that express αvβ3 and αvβ5 and α5β1 integrins. We showed here that 1a-RGD decreased cell migration and attachment, disassembled the actin cytoskeleton, reduced FAK phosphorylation, decreased the expression of target integrins at transcriptional level and induced anoikis. Our data highlight the importance of small-molecule integrin antagonists as novel tools to reduce the survival of glioma cells.

## Materials and methods

### Synthesis of 1a-RGD

**Figure 1.**
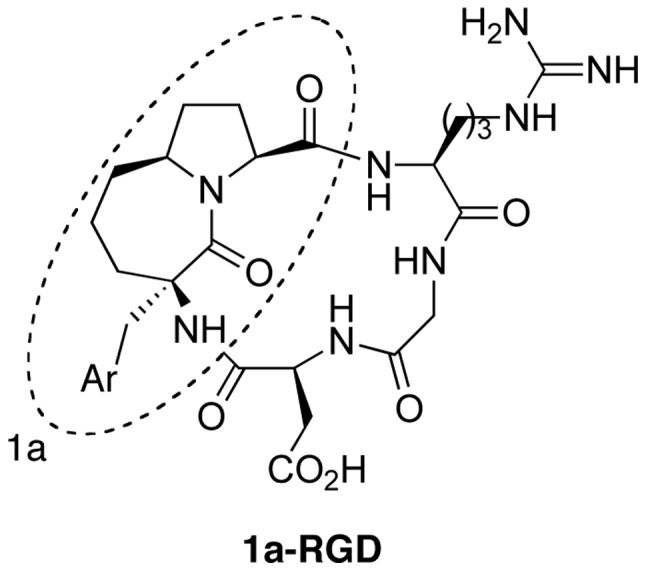
Chemical structure of 1a-RGD. The azabicycloalkane scaffold 1a is circled.

The synthesis of the cyclic-RGD derivative 1a-RGD was carried out by exploiting a solution phase method. In the first step the carboxy function of the azabicycloalkane scaffold 1a was coupled with a suitably protected NH_2_-Arg-Gly-OH dipeptide and the N-terminus of the scaffold was then coupled with an Asp residue. The resulting linear peptide sequence was finally cyclized to give the fully protected RGD-based cyclopenta peptide. The final compound 1a-RGD (Fig. 1) was purified by HPLC (8).

### Cell culture

The U251 and U373 human glioblastoma cell lines were purchased from Istituto Zootecnico Regione Lombardia (Brescia, Italy). The cell lines were grown in DMEM supplemented with 5% fetal bovine serum (FBS), 2 mM glutamine, penicillin-streptomycin (10,000 U/ml) and cells were grown at 37°C in a controlled atmosphere (5% CO_2_/95% air). Confluent cells were split (1:5–1:10 ratio) by trypsinization and used at the third-fourth passage after thawing. For all the experiments the cells were plated at a density of 10,000 cells/cm^2^. The reagents used for the cell cultures were from Euroclone, Italy.

### Cell viability assay

Cells lines plated in 96-multiwells were treated with 20 *μ*M 1a-RGD56 for 24, 48 and 72 h. At the end, 20 ml of CellTiter 96 reagent (Promega) was added to each well and after 3 h the colorimetric signal was detected by a multiwell plate reader at 490 nm. Five wells for each experimental point were used. Each experiment was repeated three times.

### FACS analysis

Cell surface αvβ3, αvβ5 and α5β1 integrin receptors were detected on fixed cells using αvβ3, αvβ5 and α5β1 Alexa 488-conjugated antibodies. Briefly, 10,000 cells for each sample were mechanically collected, fixed in 10% formaldehyde in PBS for 5 min and washed three times in PBS. Cells were incubated for 2 h at room temperature with antibodies. Samples were analyzed by a FACSCalibur instrument (Becton-Dickinson, Franklin Lakes, NJ). Each experiment was repeated three times.

To assess the onset of apoptosis the Annexin technique was used. Cells (10,000) for each sample were mechanically collected and, after washing in PBS, incubated for 5 min in the presence of Annexin and propidium iodide (PI, Sigma-Aldrich). Each experiment was repeated three times.

### BrdU-ELISA cell proliferation assay

Cells were plated in 96-multiwells in growth medium and treated with 20 *μ*M 1a-RGD for increasing time periods. At the end, 10 *μ*l of BrdU from the cell proliferation ELISA BrdU kit (Roche Diagnostics) was added to each well. After a 5-h incubation the assay was performed following the manufacturer’s instructions. The samples were evaluated using a multiwell reader at 450 nm. Eight wells were used for each experimental point and each independent experiment was repeated three times.

### Cell migration assay

Cells were plated in serum-free DMEM on a Matrigel coated Transwell (Costar). In the bottom of the well 500 *μ*l DMEM containing 10% FBS was placed as a chemo attractant. The migration assay was carried out for 12 h in the presence of 20 *μ*M 1a-RGD in the cell culture incubator. After removing the Matrigel, the cells present on the lower face of the membrane were stained using DAPI (Sigma-Aldrich) and counted using a fluorescence microscope. Cells were counted in 10 fields for each membrane.

### Adhesion assay

Cells were harvested in PBS-EDTA 5 *μ*M, resuspended in DMEM containing 5% FBS, and plated for 1 h in fibronectin-coated wells (10 *μ*g/ml) in the presence of 20 *μ*M 1a-RGD. Unattached cells were removed by two washes with PBS and attached cells were subjected to cell viability MTS assay (Promega) for quantification (9).

### Immunocytochemical analysis

For actin cytoskeleton detection, cells were plated onto polylysine-coated coverslips and incubated in the presence of 20 *μ*M 1a-RGD for 4 h. At the end of the treatments the cells were washed three times in PBS and fixed in 3.7% formaldehyde for 15 min at 37°C. The cells were then washed in PBS and incubated for 45 min in PBS containing 0.1% Triton X-100, 1% bovine serum albumin (BSA) and 10% normal goat serum. The cells were then incubated for 90 min at 37°C with Alexafluor 633 phalloidin diluted 1:7000 in PBS containing 0.01% Triton X-100 and 0.1% BSA (wash solution). At the end of the incubation, cells were repeatedly washed with PBS and counterstained with 1 *μ*M Hoechst 33342 for 15 min at room temperature. At the end of the incubation, cells were washed twice with PBS and once with distilled water. Coverslips were mounted with Mowiol. Images were acquired by laser scanning confocal microscopy, x40 oil immersion objective (TCP-SP3, Leica) and analyzed by dedicated Leica software. Each experiment was repeated at least twice.

### Western blot analysis

Cells grown in 60-mm dishes were treated for the indicated time with 20 mM 1a-RGD. The cells were then rinsed twice in ice-cold PBS and 200 ml of cell lysis buffer was added to the dishes (composition: 50 mM Tris-HCl pH 7.4, 1% v/v NP-40, 0.25% w/v sodium deoxycholate, 1 mM phenylmethylsulphonyl-fluoride (PMSF), 1 mM Na_3_VO_4_, 1 mM EDTA, 30 mM sodium pyrophosphate, 1 mM NaF, 1 mg/ml leupeptin, 1 mg/ml pepstatin A, 1 mg/ml aprotinin and 1 mg/ml microcystin). After scraping, the cells were sonicated for 10 sec, centrifuged at 12,000 × g for 5 min at 4°C and the amount of proteins in the supernatant was measured by the BCA protein assay kit (Pierce). For western blot analysis, 30 *μ*g of proteins was separated by 10% SDS-PAGE at 150 V for 2 h and blotted onto 0.22-mm nitrocellulose membranes at 50 mA for 16 h. The membranes were first blocked for 2 h in Tris-buffered saline solution (TBST composition: Tris 10 mM, NaCl 150 mM, 0.1% Tween-20) plus 5% low fat dry milk (TBSTM) and then incubated with the appropriate antibody diluted 1:1000 in TBSTM, for 16 h at 4°C under gentle agitation. The membranes were rinsed three times in TBST and then incubated for 2 h at 21°C with a goat anti-rabbit IgG horseradish peroxidase-conjugated secondary antibody (Upstate Biotechnology), diluted 1:10,000 in TBSTM. The membranes were rinsed three times in TTBS and the luminescent signal was detected by the ECL Plus Western Blotting Detection system (Amersham). Each experiment was repeated at least twice.

### ELISA apoptosis assay

For the relative quantification of apoptosis a sandwich immunoassay was performed to detect nucleosomes (Cell Death Detection ELISA, Roche Diagnostics). Cells were plated in 12-multiwell plates in growth medium and treated with 20 *μ*M 1a-RGD for increasing times. At the end of the incubation time, the assay was performed following the manifacturer’s instructions. Finally, the samples were read in a multiwell reader at 405 nm. Eight wells were used for each experiment and each experiment was repeated three times.

### Real-time quantitative PCR

The primers were designed by using the ‘Primer3 Input’ software (http://frodo.wi.mit.edu/cgi-bin/primer3/primer3.cgi/primer3_www.cgi) and the specificity of each primer was controlled by the BLAST software. Cells were treated with 20 *μ*M 1a-RGD for 24 h and total RNA was extracted. Quantitative real-time RT-PCR reactions were performed as previously reported (9). At the end of the reaction, a melting curve analysis was carried out to check for the presence of primer-dimers. Comparison of the expression of each gene between its control and stimulated states was determined with the ΔΔCt method using RPL6 as housekeeping gene (10). Experiments were performed on three different cell preparations and each run was analyzed in duplicate.

### Statistical analysis

Statistical analysis was performed by Instat3 software. Data are expressed as the means ± SD. The statistic significance values (p) are referred to control values.

## Results

### Expression of αvβ3, αvβ5 and α5β1 integrins in glioblastoma cell lines

To validate our model, preliminary experiments were performed to assess whether U251 and U373 cell lines, grown in our culture conditions, expressed the αvβ3, αvβ5 and α5β1 integrins targeted by 1a-RGD. Classic RT-PCR experiments demonstrated that U251 and U373 cells expressed mRNA for these integrin subunits (data not shown).

**Figure 2.**
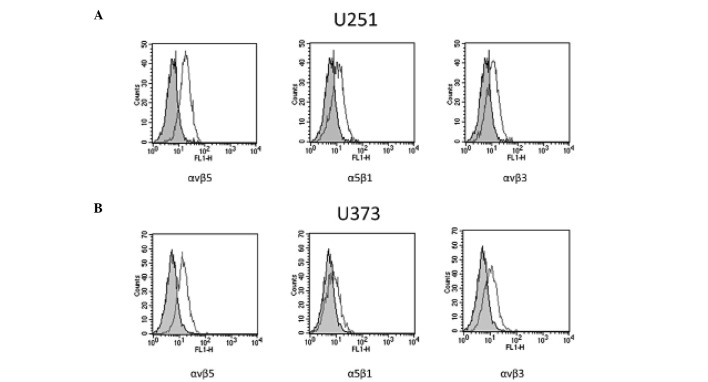
Integrin expression on the cell surface. αvβ3, αvβ5 and α5β1 integrin receptors were detected on the cell surface in the U251 and U373 cell lines by FACS analysis.

The expression and membrane localization of αvβ3, αvβ5 and α5β1 receptors were further assessed by FACS analysis. The results showed that the three receptors were detected on the cell membrane surface in the U373 and U251 cells (Fig. 2).

### 1a-RGD decreases the cell viability of glioblastoma cells

To ascertain whether 1a-RGD exerts any effect on cell growth, we first studied the effect of 1a-RGD on U251 and U373 cell viability by treating the cells with increasing concentrations of 1a-RGD. Concentration/response curves were constructed following treatment of the cells with increasing concentrations of 1a-RGD for 24, 48 and 72 h. An IC_50_ value of 10.2±0.8 *μ*M was found when cells were exposed to the compound for 72 h and this result prompted us to use 20 *μ*M 1a-RGD in the following experiments. When the U251 and U373 cells were treated with 20 *μ*M 1a-RGD for 24, 48 and 72 h, a statistically significant decrease in cell viability was observed after 72 h of treatment (data not shown).

To rule out the possibility that the observed decrease in cell viability was due to a toxic effect of 1a-RGD on the cells, LDH release at different time-points was measured. As expected, no changes in LDH release were observed even when concentrations >20 *μ*M were used, indicating that, under our conditions, 1a-RGD did not decrease cell viability by an aspecific toxic mechanism.

### 1a-RGD reduces the proliferation of glioblastoma cells

**Figure 3.**
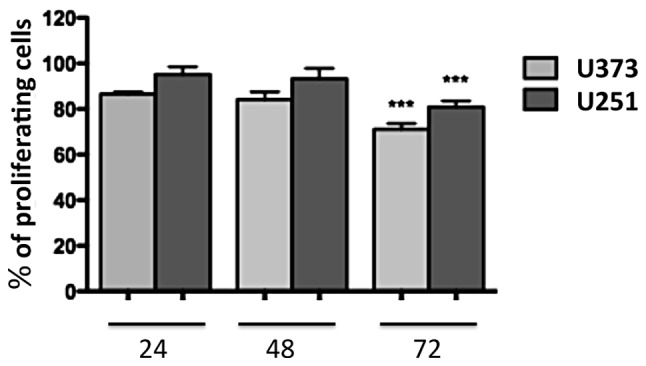
Cell proliferation assay. Cells were treated for increasing times with 20 *μ*M 1a-RGD, and a BrdU incorporation assay was performed. After 72 h a decrease in the proliferation rate was observed in both cell lines. ^***^p<0.001.

**Figure 4.**
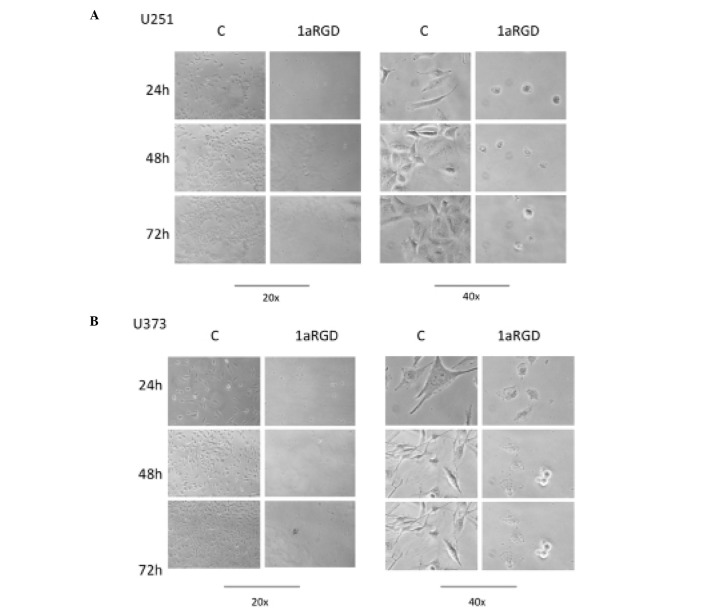
Phase contrast images. During 1a-RGD treatments, phase contrast images of treated and control cells were captured. After 72 h the cell shape was deeply altered and the majority of the cells appeared detached.

U251 and U373 cells were treated for 24, 48 and 72 h with 20 *μ*M 1a-RGD, and BrdU assays were performed to evaluate the effect of 1a-RGD on cell proliferation. A significant decrease in the cell proliferation rate was observed after 72 h (Fig. 3). During these time-course experiments, phase contrast images of the cells were captured. The images clearly display detachment and changes in cell morphology following 72 h of treatment (Fig. 4).

To confirm the reduction in the cell proliferation rate observed in BrdU experiments, cyclin D1 and p21 mRNA expression levels were measured by real-time quantitative PCR. U251 and U373 cells were treated with 1a-RGD for 72 h, and total RNA was extracted and retrotranscribed to cDNA. The treatment of U251 and U373 cell lines with 20 *μ*M 1a-RGD elicited a significant decrease in cyclin D1 mRNA and a significant increase in p21 mRNA.

**Figure 5.**
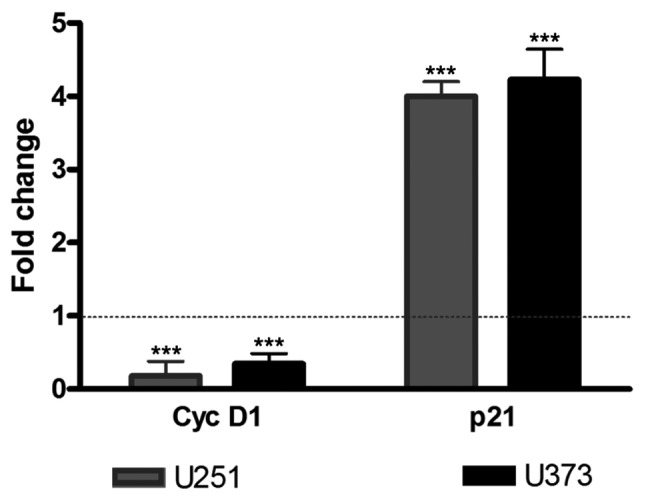
p21 and cyclin D1 mRNA expression. Cells were treated for 72 h with 20 *μ*M 1a-RGD, RNA was extracted and real-time PCR performed. A decrease in cyclin D1 and an increase in the p21 mRNA content compared to the control was observed in both cell lines. ^***^p<0.001 compared to controls.

Single transcript variations were calculated by the ΔΔCt method using RPL6 as a reference gene as reported in Materials and methods. Data are expressed as fold change and the results are summarized in Fig. 5.

### 1a-RGD reduces the adhesive capability of glioma cell lines

**Figure 6.**
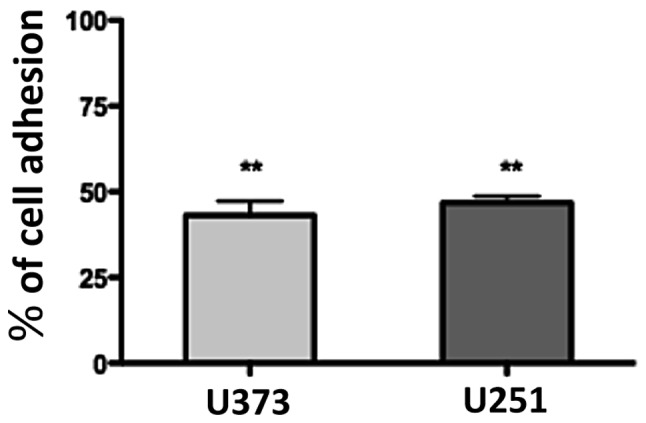
Cell adhesion assay. Cells were plated for 1 h on fibronectin-coated wells in the presence of 20 *μ*M 1a-RGD. Unattached cells were removed and attached cells were quantified by MTS assay. ^**^p<0.01 compared to controls.

To ascertain whether 1a-RGD affects cell attachment and interferes with integrin binding to cytoskeleton ECM, attachment tests were performed as described. When cells were plated on fibronectin-coated wells in the presence of 20 *μ*M 1a-RGD, 1 h after plating only ∼50% of the cells were attached compared to the controls; the quantification of attached cells was performed by an MTS assay, as described in Materials and methods (Fig. 6). This result indicates that the compound exerts a strong inhibitory effect on the cell attachment process. Similar results were obtained when the wells were coated with Matrigel.

### 1a-RGD induces cytoskeleton disassembly in glioma cell lines

**Figure 7.**
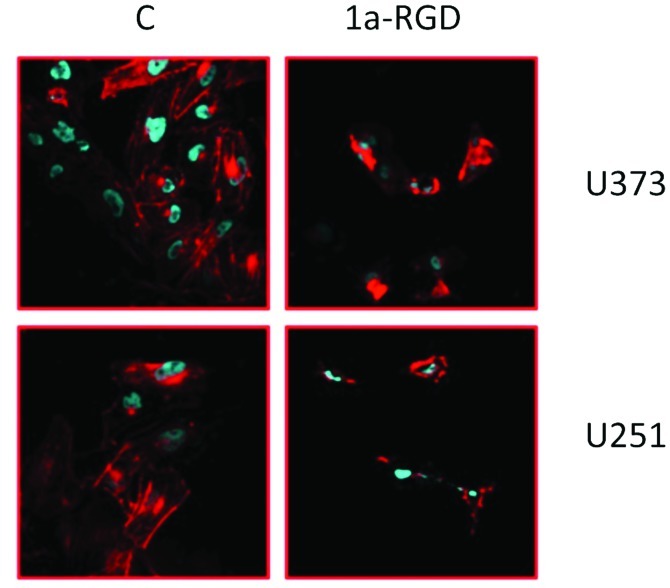
Cytoskeleton disassembly. Cells were plated onto polylysine coated coverslips and incubated in the presence of 20 *μ*M 1a-RGD for 4 h. At the end of the treatments, the cells were stained with fluorescent phalloidin and Hoechst 33342. Images were acquired with laser scanning confocal microscopy. Each experiment was repeated at least twice.

To evaluate whether the observed effects on cell adhesion are related to cytoskeleton alterations, we investigated the effect of short-term (4 h) 1a-RGD treatment on the cytoskeleton by the use of confocal microscopy. The U373 and U251 cells were plated on polylysine-coated slides and treated with 20 *μ*M 1a-RGD for 4 h. After fixing, they were marked with fluorescent phalloidin and DAPI, as described in Materials and methods. The results showed that the compound induced structural cytoskeletal disassembly. Actin fibers appeared ruffled and the cell shape was deeply affected (Fig. 7). These data indicate that 1a-RGD, by antagonizing integrins binding to the well, caused marked changes in the cytoskeletal structure.

### 1a-RGD inhibits the cell migration of glioma cell lines

**Figure 8.**
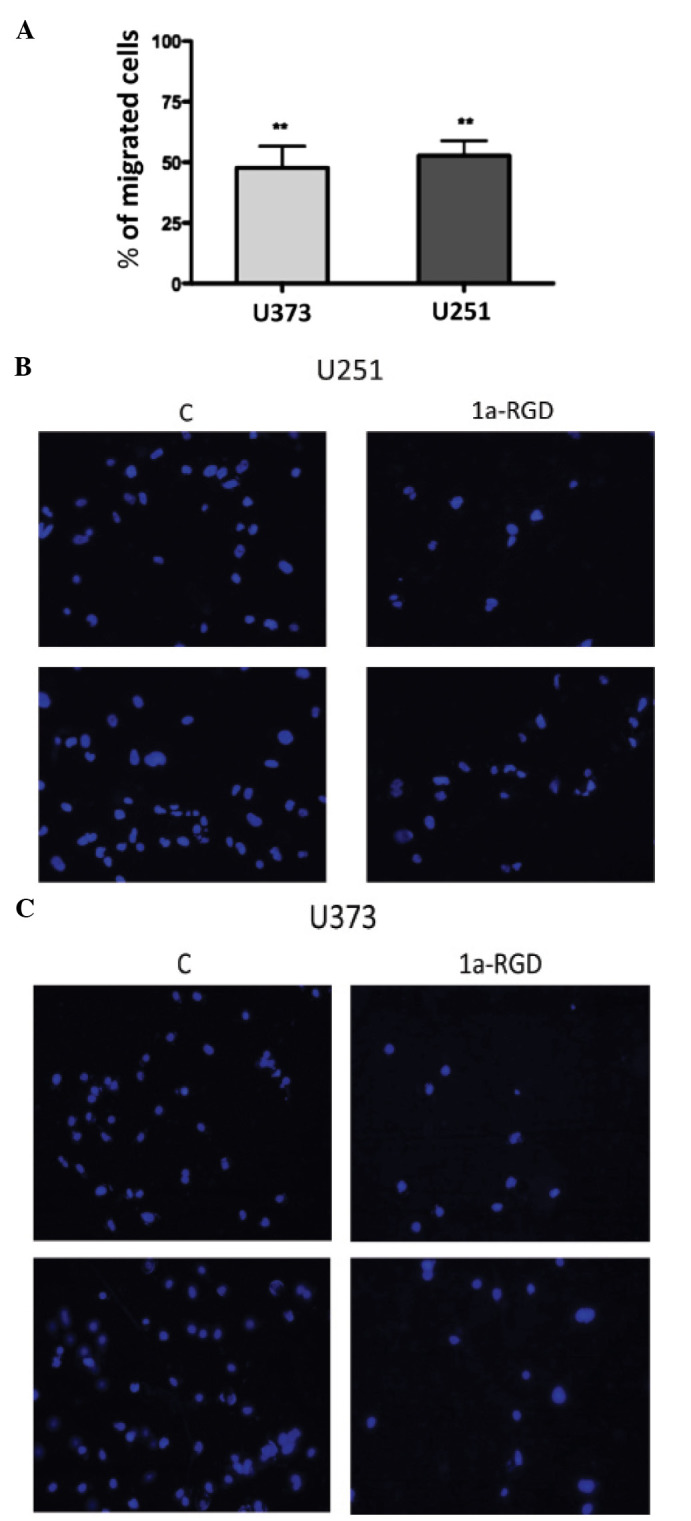
Cell migration assay. Cells were plated in serum-free DMEM on a Matrigel-coated Transwell. In the bottom of the well, 500 *μ*l DMEM containing 10% FBS was placed as a chemoattractant. The migration assay was carried out for 12 h in the presence of 20 *μ*M 1a-RGD. After removing the Matrigel, the cells present on the lower surface of the membrane were stained with DAPI and counted using a fluorescence microscope and ≥10 fields were counted for each membrane. (A) Results reported in the graph are expressed as a percentage of migrated cells (^**^p<0.01 compard to controls). (B and C) Representative images of the lower surface of the membrane showing migrated cells stained with DAPI.

The cellular machinery that is activated during cell motility and cell migration processes requires a functional cytoskeleton (11). The observation that 1a-RGD treatment induces changes in actin fibers, led us to investigate the effect of this compound on cell motility by migration assays as described in Materials and methods. The U373 and U251 cells were plated on a Matrigel- coated membrane in serum-free medium in the presence of 20 *μ*M 1a-RGD for 12 h. At the end of the treatment, the cells on the lower side of the membrane were stained with DAPI and counted (see Materials and methods). The results, expressed as a percentage compared to the control, showed a marked decrease in cell number on the lower side of the membranes in the presence of 1a-RGD (Fig. 8A); representative fields of the lower membrane sides are shown in Fig. 8B and C.

### 1a-RGD inhibits FAK phosphorylation but not AKT and ERK phosphorylation

**Figure 9.**
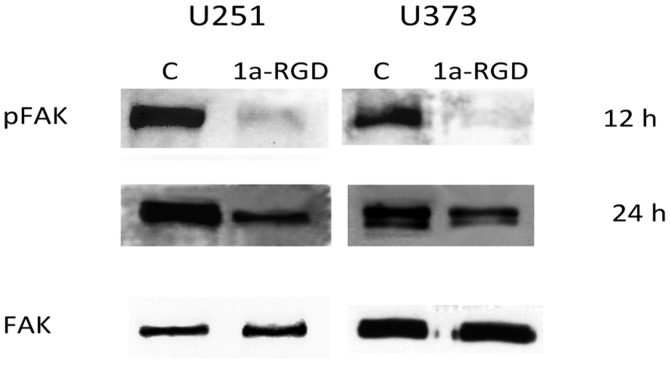
Western blot analysis of FAK phosphorylation. U251 and U373 cells were treated for the indicated times with 20 *μ*M 1a-RGD, and cell extracts were subjected to western blotting using specific antibodies. A marked increase in phosphorylated FAK was detected in 1a-RGD-treated cells. Experiments were repeated at least three times.

The tyrosine kinase FAK resides in focal adhesions formed by integrin clustering and is functionally linked to integrin activation. As 1a-RGD appears to interfere with cell attachment, cell migration and cytoskeleton assembly, we evaluated the effect of this antagonist on FAK phosphorylation. Western blot experiments were performed as indicated above (Fig. 9). In both cell lines, a marked decrease in pFAK signal is detected after 12 h of treatment and the signal was downregulated even after 24 h. These data confirm that 1a-RGD binding to integrin receptors inhibits cellular processes required for cell attachment and cell migration.

**Figure 10.**
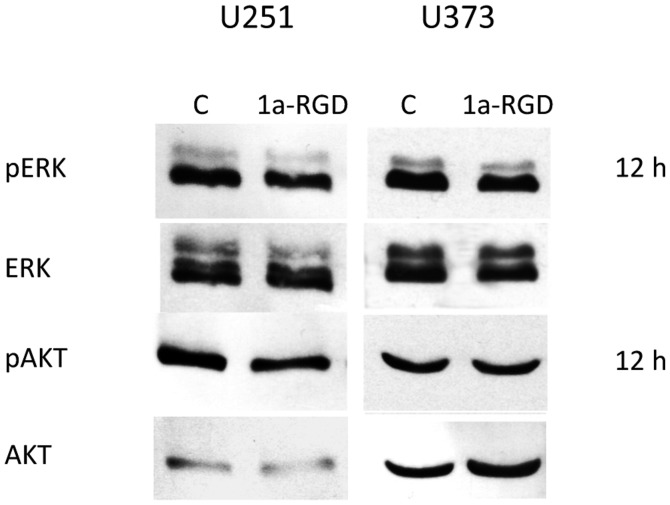
Western blot analysis of ERK and AKT phosphorylation. U251 and U373 cells were treated for 12 h with 20 *μ*M 1a-RGD, and cell extracts were subjected to western blotting using specific antibodies. No effect on ERK and AKT phosphorylation was detected in the 1a-RGD-treated cells. Experiments were repeated at least three times.

Western blot experiments were performed to further investigate the effect of 1a-RGD treatment on integrin-linked signal transduction. Cells were treated with the compound at different time points. After 72 h of treatments, no effect on Akt or ERK phosphorylation was observed in both cell lines (Fig. 10).

### 1a-RGD induces anoikis in glioma cell lines

**Figure 11.**
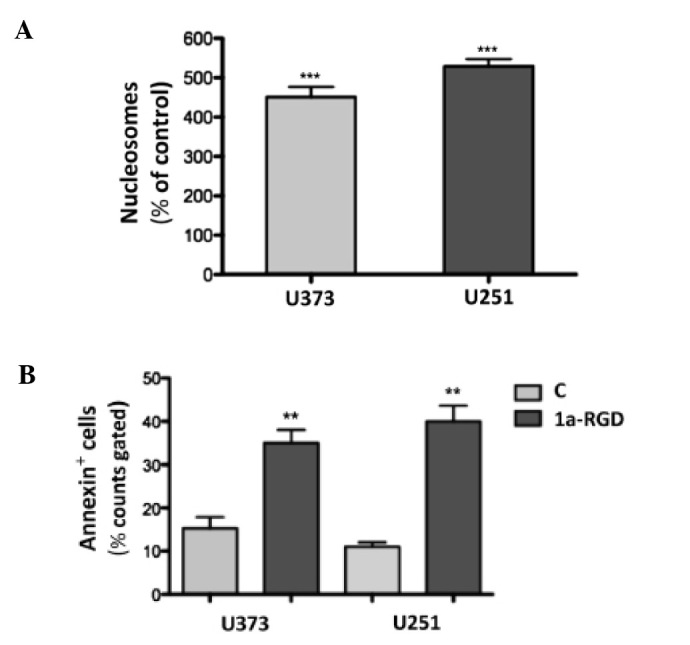
Anoikis detection. (A) Cells were treated for 72 h with 20 *μ*M 1a-RGD and an ELISA assay for nucleosome detection was performed. A marked increase in nucleosome content was observed. Data are expressed as a percentage of the controls ± SD. ^***^p<0.001 compared to controls. (B) Cells were treated with 20 mM 1a-RGD for 72 h. Cells (10,000) for each sample were collected, stained by Annexin and propidium iodide and analyzed by FACS. ^**^p<0.01 compared to controls.

In order to assess whether the 1a-RGD treatment induces apoptosis, cells were treated with 20 *μ*M 1a-RGD for increasing times and then cell death was assessed by nucleosome assay. After 72 h, 1a-RGD induced a significant increase in fragmented DNA (Fig. 11A). Similar results were obtained when cell death was measured by Annexin V staining (Fig. 11B).

FACS experiments were also separately performed on attached and detached cells from the same wells treated with 20 *μ*M 1a-RGD for 72 h. Notably, Annexin-positive cells were almost exclusively found in the detached fraction.

### 1a-RGD reduces mRNA expression of the α_5_ and β_1_ integrin subunits

It is known that, in some cases, long term occupation of cell surface receptors by specific antagonists may induce compensatory and adaptive changes of their expression at the transcriptional level by complex positive or negative feedback mechanisms. Since in the literature no data concerning the long-term effects of RGD-antagonist treatment on integrin expression are available, we decided to evaluate whether 1a-RGD modifies mRNA levels of α_5_ and β_1_ integrin subunits in long-term experiments. U251 and U373 cells were treated with 20 *μ*M 1a-RGD for 72 h, total RNA was extracted, retrotranscribed to cDNA and real-time PCR was performed. Data were obtained by the ΔΔCt method using RPL6 as reference gene (Table I). In both cell lines, while the amount of α_v_, β_3_ and β_5_ mRNA was not changed, a statistically significant decrease in α_5_ and β_1_ mRNA compared to the control (=1) was found: U373 α_5_ 0.51±0.03 (p<0.01); U373 β_1_ 0.66±0.04 (p<0.05); U251 α5 0.60±0.03 (p<0.05); U251 β1 0.41±0.02 (p<0.01).

## Discussion

The metastatic cascade of tumor cells involves several sequential steps: detaching from the original tumor mass, migration to distant sites and attachment to the tumoral niche (12). During the steps of metastasis formation, cancer cell survival depends on the ability of cells to interact with ECM proteins and integrins regulate both migration and adhesion processes. For this reason, preventing tumor cells binding to the ECM is an important strategy to inhibit cancer cell spreading and induction of anoikis. Small-molecule integrin antagonists bearing the RGD sequence, such as the prototype compound cilengitide, have been shown to exert antiangiogenic and anti-proliferative activity in glioma therapy (13).

However, the mechanisms underlying the antiangiogenic and antiproliferative effects of small-molecule integrin antagonists are still unclear.

Preclinical studies in patients with recurrent GBM have shown that cilengitide monotherapy inhibits glioblastoma growth with modest antitumor activity (6) and, in combination with radiotherapy, decreases tumor proliferation (7). However, a recent study has raised concern about the use of cilengitide monotherapy in glioma demonstrating that, under certain experimental conditions and at nanomolar concentrations, this molecule promotes rather than inhibits angiogenesis (14).

Considerable efforts have been made to synthesize new RGD compounds with anticancer activity and 1-aRGD belongs to a family of new RGD compounds synthesized in our laboratory (15).

In a main attempt to characterize the functional effects elicited by 1a-RGD in human glioma cell lines grown *in vitro*, we found that 1a-RGD, although it induces modifications in cell functionality already described for other similar compounds, nevertheless it displays certain novel features not previously reported for other RGD integrin antagonists.

The ability of 1a-RGD to decrease adhesion and proliferation in glioma cell lines are in excellent agreement with the effects elicited by the small α5β1 integrin antagonist SJ749 that dose-dependently inhibits adhesion to fibronectin and proliferation in the glioma cell lines A172 and U87 (16).

In a similar study, an RGD peptide that potently binds to the αvβ3, αvβ5 and α5β1 integrins was found to decrease the proliferation rate and adhesion in a panel of rat and human glioma cells (17).

Another study reported that in glioma cell lines cilengitide induces detachment and cell death with only a modest effect on cell viability and without appreciable perturbation on cell migration and invasivenesss (18).

Our data indicate that the main triggering event underlying the observed cellular effects elicited by 1a-RGD was the inhibition of cell attachment via direct antagonism with endogenous ECM ligands towards target integrins as demonstrated by cytoskeleton disassembly and inhibition of focal adhesion kinase FAK. However, under our conditions, 1a-RGD markedly affected not only cell attachment but also cell migration, and FAK inhibition appears to be an important step in this process.

FAK is the link between integrins and downstream signaling transduction pathways and activates ERK and PI3K/AKT pathways involved in cell proliferation and migration (19); short term cilengitide treatment inhibited the FAK/Src/AKT-dependent pathway in endothelial and glioma cells but did not affect ERK activation in HUVEC cells (20). In partial contrast with this observation, we found a significant FAK phosphorylation decrease in cells treated up to 24 h with 1a-RGD but no appreciable effect on AKT and ERK phosphorylation was observed. The most likely explanation for this discrepancy is that other receptor systems, merging on the AKT- and ERK-dependent pathways, may likely overcome the weak inhibitory effect elicited by 1a-RGD.

The concept that the pattern of integrin expression affects cell migration has been demonstrated in some cancer cell types. In melanomas, expression of αvβ3 integrin correlates with tumor invasion (21) and overexpression of αvβ3 is reported to mediate the migratory and invasive phenotype of imatinib-resistant adherent chronic myelogenous leukemia cells (22). In addition integrin β5 expression plays a critical role in breast carcinoma cell migration and enhances their proliferative capacities (23).

We found that prolonged treatment with 1a-RGD induced, at the transcriptional level, a downregulation of α5 and β1 subunits in glioma cell lines and this event is likely to contribute to the reduction in cell migration. The capability of decreasing the expression of selected target integrins by pharmacological treatment is quite intriguing as α5β1 integrin, together with concomitant EGFR activation, is required for mutated p53 driven enhancement of cancer cell motility (24).

The combined reduction in cell migration and attachment are critical factors in reducing cancer cell dissemination together with their ability to form new peritumoral niches in the brain. These effects elicited by 1a-RGD have crucial implications for the management of glioma recurrence since the grim prognosis of glioma can be mainly ascribed to the ability of a subpopulation of cancer cells to disseminate throughout the brain giving rise to new local solid mass reformation. Migrating non-adherent cells undergo anoikis but metastatic cells acquire resistance to anoikis and therefore the search for new proapoptotic drugs is the most important unsolved problem in oncology.

Although widely investigated in several cancer cell lines, few studies have reported a proapoptotic effect of small-molecule RGD integrin antagonists in glioblastoma cells. However, due to their limited efficacy in monotherapy in recent years, other approches have been devised to enhance the induction of cell death elicited by small-molecule integrin antagonists with the purpose to improve their clinical relevance. For example, these molecules could amplify the proapoptotic effect of other anticancer drugs, since in glioma cells the co-administration of two selective α5β1 integrin inhibitors was found to facilitate cell apoptosis induced by two currently used chemotherapeutic agents, ellipticine and temozolomide, in brain tumor therapy (25). Furthermore, in endothelial cells, the administration of the selective αvβ3 and αvβ5 antagonist RGDfV was found to potentiate the apoptotic cell death induced by the c-Abl kinase inhibitor STI-571 (26).

In addition, novel RGD-like compounds could be screened as shuttle molecules when chemically linked to antitumoral drugs to improve local bioavailability and to reduce the dose sufficient for cell killing. In MCF-7 breast cancer cells expressing αvβ3 integrin, the integrin antagonist cyclic-RGD-bioshuttle functionalized with the antitumoral drug temozolomide (TMZ) displayed a 10-fold decrease in the IC_50_ when compared to the underivatized TMZ (27). The co-administration or chemical coupling of these inhibitors with clinically relevant drugs deserves further testing first in different *in vitro* models and warrants additional *in vivo* studies in animal experimental models.

One serious limitation of this study is that the effects exerted by 1a-RGD have been detected in glioma cell cultures propagated for a long period in cultures that may not mirror the real genotype of the original tumor. To overcome this pitfall, the functional cellular effects elicited by 1a-RGD reported here must be tested in a more reliable *in vitro* cell model that more closely resembles the phenotype of glioma cells *in situ*. The recent characterization of glioma cancer stem cells, that can be grown either attached to a laminin substrate or in suspension as neurospheres in the absence of serum and that appear like true precursors of circulating metastatic cells (28), could represent a suitable alternative experimental *in vitro* model to shed new light on this promising avenue of research.

In conclusion, we provide new insights into the functional cellular effects induced by a novel small-molecule RGD integrin antagonist in human glioblastoma cell lines that can potentially improve the pharmacological approach and clinical management of glioblastoma chemotherapy.

## Figures and Tables

**Table I.** Primer sequences and PCR product size (bp).

αv	NM_002210	F: actggcttaagagagggctgtg	110
		R: tgccttacaaaaatcgctga	
β3	NM_000212	F: agacactcccacttggcatc	123
		R: tcctcaggaaaggtccaatg	
β5	NM_002213	F: agcctatctccacgcacact	
		R: cctcggagaaggaaacatca	91
α5	NM_002205	F: cctgctgtccaccatgtcta	
		R: ttaatggggtgattggtggt	138
β1	NM_133376	F: tccaatggcttaatttgtgg	
		R: cgttgctggcttcacaagta	190
CycD1	NM_053056	F: ctcacgcttacctcaaccatc	
		R: ctttggcctctcgatacacac	130
p21	NM_000389	F: atatggggctgggagtagttg	
		R: ccaggccagtatgttacagga	132
RPL6	NM_001024662.1	F: agattacggagcagcagcgcaagattg	
		R: gcaaacacagatcgcaggtagccc	105
